# Enhancing organizational health literacy through pediatric clinical training: Mini-CEX–based formative assessment in case-based learning for pediatric pneumonia—a perspective

**DOI:** 10.3389/fpubh.2026.1880936

**Published:** 2026-07-08

**Authors:** Sheng-nan Ge, Bing Li, Bing-yan Mu, Li-sheng Wan

**Affiliations:** 1 Department of Pediatric, Luohu District Hospital of Traditional Chinese Medicine, Shenzhen, China; 2 Department of Chinese Medicine, Shenzhen Children’s Hospital, Shenzhen, China

**Keywords:** case-based learning, clinical training, formative assessment, health equity, Mini-clinical evaluation exercise, organizational health literacy, pediatric pneumonia

## Abstract

Organizational health literacy (OHL) remains a critical yet underexplored dimension of health equity, particularly at the interface between clinical training and patient care. This article proposes a conceptual reframing of the Mini-Clinical Evaluation Exercise (Mini-CEX) as an OHL-oriented formative assessment tool within case-based learning for pediatric pneumonia. The OHL-Mini-CEX model integrates three assessment domains—teach-back evaluation, de-jargonization capacity, and empowering guidance—to systematically evaluate residents’ communicative competence in addressing health literacy barriers. We delineate the mechanisms through which this instrument operates: embedding equity-conflict scenarios into teaching cases, generating bidirectional formative feedback for both clinicians and organizations, and constructing a closed loop from micro-level training to macro-level health equity outcomes. Implementation challenges, including faculty preparedness gaps and psychometric rigor, are examined alongside integrated strategies drawing on public health collaboration. This perspective advances a theoretically grounded and practically actionable pathway for transforming clinical education into an institutional driver of health equity.

## 1 Introduction

### 1.1 Dual challenge of health equity and organizational health literacy (OHL)

Childhood pneumonia offers an incisive lens for examining structural barriers to health equity (1, 2). As the leading infectious cause of death among children under five worldwide, pneumonia displays stark outcome disparities across socioeconomic, ethnic, and geographic lines (1). Attributing these disparities solely to pathogen variation or unequal access to medical technology overlooks a deeper problem: the functional breakdown at the interface between healthcare systems and families (2, 3). Too often, health information fails to reach its intended recipients in forms that are comprehensible, actionable, and culturally appropriate. It is at this critical juncture that deficits in OHL magnify health inequities (3).

OHL denotes an institution’s capacity to equitably enable individuals to find, understand, and use health information and services for informed decision-making (2, 3). This concept shifts responsibility from individual deficits to system-level design, processes, and culture (2, 4). In principle, OHL provides a viable framework for narrowing outcome gaps in conditions such as childhood pneumonia (3). In practice, however, interventions have largely defaulted to a “front-end simplification” approach—channeling resources into improving the readability of written forms, consent documents, and navigational signage—while leaving the most consequential element, the clinical conversation, largely unaddressed (2).

### 1.2 Absent role of clinical education and an emerging opportunity for transformation

This neglect exposes a significant structural gap: the absence of clinical education from the OHL agenda (2, 5). Frontline clinicians, particularly pediatric residents still shaping their professional competencies, function as the frontline implementers of OHL (5). Yet current training systems have not systematically equipped them for this role (6, 7). Their routine responsibilities—history-taking, shared decision-making, discharge education—constitute the final and most critical step in transmitting organizational health information (3, 6). If the communicative and educational skills required at this point are not embedded as integral components of institutional workflow, then upstream administrative simplifications remain isolated, superficial fixes (2, 5, 6).

We therefore propose that strengthening OHL in pediatric settings requires extending organizational efforts beyond administrative redesign to include communication- focused clinical training. The communicative competencies essential to this task—systematic identification of low health literacy, standardized use of teach-back techniques, consistent deployment of empathetic, plain-language communication—should cease to be treated as desirable personal attributes (8, 9). Instead, they should be integrated as core performance indicators within residency education and as mandatory steps within organizational care protocols. Only then can OHL be internalized as an institutionalized organizational capability rather than remaining an external graft.

## 2 Theoretical framework reconceptualized: reframing Mini-Clinical Evaluation Exercise (Mini-CEX) as an assessment tool for OHL

### 2.1 Limitations of classic Mini-CEX in pediatric pneumonia education

The Mini-CEX is a foundational formative assessment tool in competency-based medical education (10, 11). Its traditional evaluative dimensions emphasize the completeness of history-taking, the technical proficiency of physical examination, and the diagnostic accuracy of clinical reasoning (10). In case-based learning (CBL) for pediatric pneumonia, this framework effectively captures a resident’s biomedical reasoning ability. Yet it harbors a pronounced structural blind spot. Standard rating scales systematically overlook a set of communication practices with decisive implications for patient outcomes (10, 12). These include explaining the clinical logic of empiric antibiotic therapy to anxious caregivers in accessible language; verifying that families can reliably recognize danger signs of clinical deterioration, such as worsening tachypnea, nasal flaring, or feeding difficulties; and using structured communication to reduce non-urgent emergency revisits that stem from information gaps (13, 14). These routinely omitted dimensions represent the core operational elements of OHL within clinical micro-interactions (13, 14).

In addition to its widespread adoption in competency-based medical education, the Mini-CEX offers several advantages that make it particularly suitable for OHL-oriented assessment (15, 16). First, it is based on direct observation of authentic clinical encounters, enabling evaluators to assess communication behaviours in real-world contexts (16, 17). Second, its familiarity among clinical educators facilitates implementation without major curricular restructuring (17, 18). Third, its emphasis on immediate formative feedback aligns closely with the developmental nature of health literacy–sensitive communication skills (16–19). These characteristics provide a practical foundation for integrating OHL competencies into routine residency assessment.

### 2.2 Constructing an OHL-oriented Mini-CEX framework

To address these limitations, we propose the “OHL-Mini-CEX” conceptual model. This model expands the conventional assessment scale by systematically embedding the universal precautions of OHL, elevating communicative competence from an implicit expectation to an observable and measurable educational indicator (5, 20). The model introduces three core assessment domains. The first, teach-back evaluation, examines whether residents, when delivering discharge instructions for children with pneumonia, verify caregivers’ understanding of medication regimens, return precautions, and home care essentials through a non-interrogative approach and correct identified misunderstandings in a closed-loop manner (14, 21). The second, de-jargonization capacity, assesses residents’ ability to translate high-barrier clinical terms into concrete, everyday descriptions—converting “rhonchi” into “the lungs sound as though there is mucus collected inside,” or “intercostal retractions” into “the space above the breastbone and between the ribs sinks in when the child breathes”—thereby ensuring the information remains semantically accessible (12, 22). The third, empowering guidance, evaluates whether residents design family care action plans with low behavioral thresholds and strong contextual adaptability during case discussions (14, 21). This involves refining broad instructions such as “ensure adequate fluid intake” into specific, actionable steps like “offer two small spoonfuls of warm water every half hour,” effectively translating medical knowledge into routine family practice (21, 22). Through this reconceptualisation, the Mini-CEX may serve as a systemically oriented assessment instrument aligned with health equity goals (Table 1). We emphasise that this proposed function remains conceptual and requires future empirical validation regarding its educational and organisational effects.

**Table 1:** Comparison between conventional Mini-CEX and OHL-Mini-CEX in pediatric pneumonia training.

Dimension	Conventional Mini-CEX	OHL-Mini-CEX
Core educational orientation	Focuses primarily on biomedical competence and clinical reasoning	Integrates biomedical competence with organizational health literacy and health equity principles
Primary assessment objective	Evaluates individual clinical performance	Evaluates both communicative competence and equity-sensitive care delivery
History-taking assessment	Emphasizes symptom completeness and diagnostic relevance	Includes identification of social-contextual and health literacy barriers
Communication evaluation	Assesses general interpersonal communication skills	Assesses structured, health literacy–responsive communication strategies
Language use	Medical terminology commonly accepted if clinically accurate	Requires systematic de-jargonization and plain-language translation
Teach-back application	Usually not explicitly evaluated	Teach-back verification is a core assessment domain
Discharge education	Broad and experience-dependent	Structured, actionable, and comprehension-verified
Family engagement	Limited evaluation of caregiver understanding	Actively evaluates caregiver comprehension and participation
Clinical decision-making	Primarily biomedical and disease-centered	Integrates biomedical reasoning with contextual adaptability
Assessment of home-care guidance	Often generalized and non-standardized	Requires low-threshold, behaviorally actionable instructions
Evaluation framework	Individual learner-centered	Individual–organizational dual feedback framework
Feedback mechanism	Primarily supports resident learning	Supports both resident improvement and organizational learning
Data utilization	Used mainly for trainee assessment	Generates organizational quality-improvement data
Organizational function	Limited educational role	Functions as an operational tool for organizational health literacy
Relationship to health equity	Indirect and implicit	Explicitly aligned with health equity promotion
Expected long-term outcome	Improved clinical competence	Improved communication quality, caregiver adherence, and equity-sensitive pediatric care

OHL, organizational health literacy; Mini-CEX, Mini-Clinical Evaluation Exercise.

## 3 Mechanism integration: pathways from OHL-Mini-CEX under CBL mode to health equity promotion

### 3.1 Scenario embedding through pediatric pneumonia cases

Advancing health equity through clinical education demands deliberate intervention at the instructional design level (23). In this perspective article, equity-conflict scenarios refer to clinical situations where social determinants of health, limited health literacy, language barriers, cultural factors, or resource constraints create tension between evidence-based recommendations and what is realistically achievable for patients and families; in such scenarios, clinicians must adapt their communication and management strategies while preserving quality of care (24–27). In this study, we embed social-contextual variables with equity-conflict attributes directly into CBL materials for pediatric pneumonia, creating authentic clinical decision-making tensions. The teaching cases situate typical pneumonia presentations within composite backgrounds: a primary caregiver who is a mother with limited health literacy, a dialect-based communication barrier, and a damp, crowded home environment lacking access to nebulization therapy (12, 14). This design deliberately moves social determinants from the instructional periphery to the center, requiring residents to integrate biomedical and social-structural reasoning simultaneously (23). The OHL-Mini-CEX assessment anchors are then aligned with these conflict scenarios. Evaluators observe whether residents identify the social obstacles present and adjust their management plans accordingly—for instance, by providing an equivalent alternative when nebulization is unavailable, or by adopting communication strategies suited to the caregiver’s linguistic capacity (12, 21). The goal is to produce accessible care plans genuinely congruent with the patient’s social context.

### 3.2 Dual formative feedback mechanism

The formative assessment function embedded in the OHL-Mini-CEX operates through a bidirectional empowerment structure (5, 28). At the provider level, evaluators deliver structured feedback to residents immediately after each exercise, focusing on the actionability of their clinical instructions and the contextual fit of their communication strategies (21, 29). This real-time calibration prompts residents to recognize the cognitive demands their information delivery imposes on recipients and to gradually internalize a communication approach anchored to the recipient’s comprehension level (29). At the organizational level, the qualitative data accumulated across multiple OHL-Mini-CEX encounters—particularly the systematic documentation and attribution analysis of communication breakdowns—provide a rich learning repository (5, 30). For instance, if repeated assessments reveal that residents consistently fail to verify caregiver understanding of discharge instructions using teach-back techniques, departmental education committees can review these aggregated findings, identify recurrent communication barriers, and implement targeted interventions (9, 18). Such interventions might include redesigning discharge materials with plain language, developing visual educational aids, or introducing focused faculty development workshops to enhance resident communication competencies (31). These frontline educational data can directly inform system-level improvements initiatives (14). The assessment tool thus moves beyond individual evaluation, becoming a continuous data source that drives organizational learning and iterative process refinement (5, 30).

### 3.3 Logical closed loop from micro-level teaching to macro-level health equity

**Figure 1:**
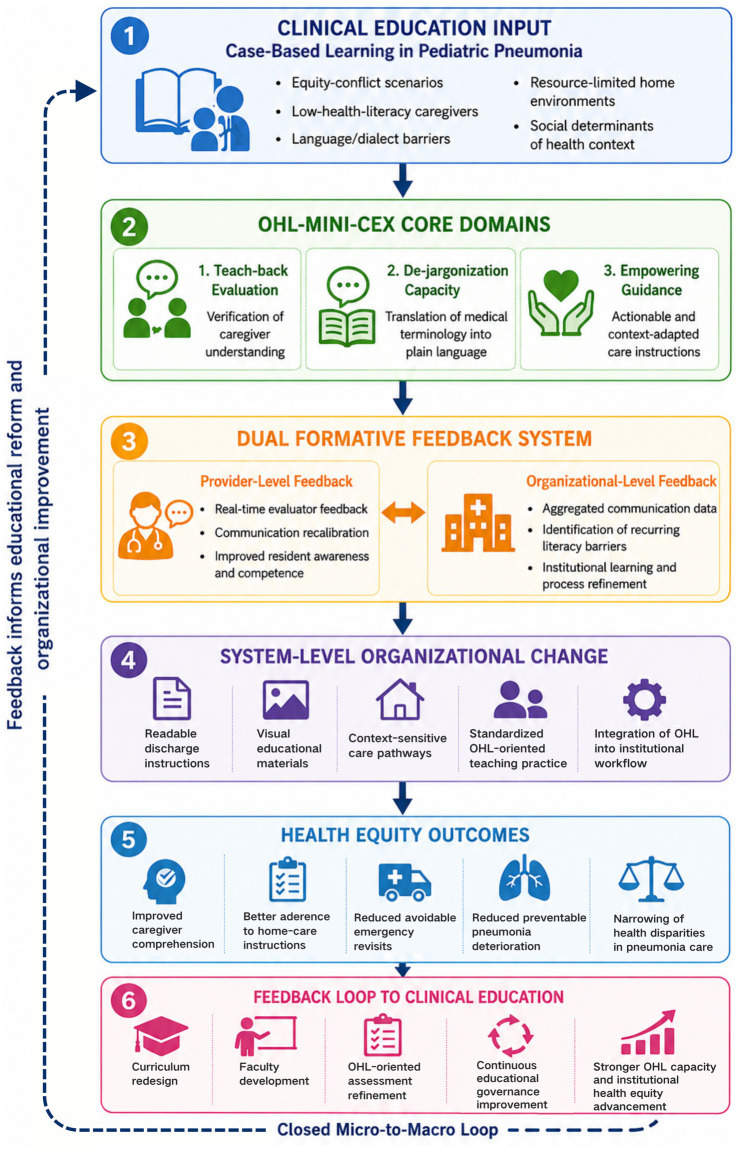
Conceptual closed-loop framework of the OHL-Mini-CEX model in pediatric pneumonia training.

The synergy of these dual mechanisms is conceptualized as a theoretical closed-loop pathway extending from micro-level educational intervention to macro-level health equity outcomes. As illustrated in Figure 1, the framework progresses through four interconnected stages: (1) educational encounters grounded in equity-conflict scenarios; (2) OHL-oriented Mini-CEX assessments with formative feedback; (3) systematic aggregation of assessment findings to inform organisational learning and quality improvement; and (4) potential long-term contributions to health equity. The model is intended to depict a theoretical pathway rather than a confirmed causal sequence. The causal relationships proposed within this framework remain hypothetical and should be interpreted as testable propositions rather than established effects. Systematically embedding OHL-infused assessment into residency training for childhood pneumonia—a high-burden condition—aims to cultivate a generation of pediatric clinicians with heightened awareness of health equity (32). Upon entering independent practice, this acquired capacity translates into effective communication with low-health-literacy families, substantially reducing disease progression and avoidable complications arising from caregiver misunderstanding of medical instructions (14, 33). In this way, the distorting effects of social determinants on pneumonia outcomes are partially mitigated at the level of clinical interaction (33). When such practices are institutionalized and scaled across an organization, the commitment to health equity descends from abstract value statements into operational, evaluable, and iterable daily practice (5, 34). This proposed closed loop offers a theoretically coherent framework for understanding how medical education reform may contribute to health equity promotion (Figure 1). Future multicenter implementation studies are needed to evaluate whether improvements in OHL-oriented assessment translate into measurable organizational and patient-level outcomes.

## 4 Challenges and strategies for advancing OHL-Mini-CEX in teaching hospitals

### 4.1 Practical barriers to implementation

Translating the OHL-Mini-CEX from a theoretical model into routine practice within teaching hospitals encounters several interlocking obstacles (2, 20). The first and most immediate is a gap in faculty preparedness. Most clinical preceptors were trained in biomedically centered systems and lack both systematic awareness of health literacy assessment and relevant training experience (2, 12, 35). As a result, the OHL-Mini-CEX risks being narrowly interpreted during implementation—its multidimensional evaluation reduced to a generic score for interpersonal communication, thereby stripping away its distinctive health equity orientation (2). The second challenge concerns the psychometric properties of the assessment scale. Dimensions central to OHL—such as the adequacy of de-jargonization or the appropriateness of empowering guidance—are inherently susceptible to evaluator subjectivity (20, 21). Without a clearly specified Behaviorally Anchored Rating Scale that operationally defines each scoring level, inter-rater reliability remains difficult to secure, undermining both the fairness of evaluation and the effectiveness of instructional feedback (20, 30).

### 4.2 Integrated strategies drawing on public health and clinical collaboration

To address these challenges, we propose three strategies that integrate public health perspectives with clinical teaching practice (Table 2). First, establish a collaborative faculty development mechanism. We recommend pairing clinical preceptors with public health professionals in structured teaching partnerships (32, 34). Through joint seminars and reciprocal observation of assessments, both sides can calibrate shared criteria for “information comprehensibility” and “plan actionability,” bridging the gap between clinical reasoning and population health perspectives (5, 20). Second, pursue a digital empowerment approach. Embedding health literacy decision-support prompts within the electronic Mini-CEX platform can compensate for initial gaps in faculty experience (28, 30). At critical assessment junctures—for instance, during the discharge counseling segment—a reminder window could automatically appear, prompting the evaluator to confirm whether the resident has completed a teach-back verification (12, 21). Such prompts reduce the risk of assessment omissions while faculty competence is still being cultivated (20, 28). Third, construct a systemic integration pathway. We propose incorporating de-identified, aggregated OHL-Mini-CEX data into departmental annual OHL reports. This mechanism converts process data from teaching assessments into measurable indicators of the organization’s health equity performance (2, 20). It consolidates communication practices that would otherwise remain scattered across individual teaching encounters into trackable, comparable, and iterable organizational assets, thereby advancing teaching hospitals from experience-based preceptorship toward evidence-informed, equity-oriented educational governance (5, 36, 37).

**Table 2:** Practical challenges and integrated strategies for implementing the OHL-Mini-CEX framework in teaching hospitals.

Implementation challenge	Underlying structural problem	Proposed integrated strategy	Expected educational and organizational impact
Faculty preparedness gap	Many clinical preceptors lack systematic training in organizational health literacy and equity-oriented communication assessment	Establish collaborative faculty development programs pairing clinical educators with public health professionals	Promotes shared assessment standards and strengthens faculty competency in OHL-oriented evaluation
Reduction of OHL assessment to generic communication scoring	Evaluators may overlook the distinct equity-oriented dimensions of OHL-Mini-CEX	Develop structured evaluator training emphasizing teach-back, de-jargonization, and empowering guidance domains	Preserves the conceptual integrity of the OHL-Mini-CEX framework
Subjectivity in scoring	OHL-related dimensions are vulnerable to evaluator interpretation variability	Introduce Behaviorally Anchored Rating Scales with operationalized scoring criteria	Improves inter-rater reliability, fairness, and psychometric rigor
Inconsistent teach-back verification	Faculty may omit critical communication checkpoints during assessment	Embed digital prompts and automated reminders within electronic Mini-CEX platforms	Enhances assessment completeness and reinforces standardized evaluation behaviors
Limited organizational learning from educational encounters	Communication failures remain fragmented across isolated teaching interactions	Aggregate de-identified OHL-Mini-CEX data into institutional quality-improvement systems	Converts frontline educational observations into organizational learning assets
Weak integration between clinical education and public health perspectives	Clinical teaching often prioritizes biomedical reasoning while neglecting social determinants of health	Develop interdisciplinary educational partnerships involving clinicians, public health educators, and health literacy specialists	Encourages integration of biomedical and social-contextual reasoning
Lack of contextual adaptability in resident guidance	Standard discharge instructions may not match caregiver literacy levels or living conditions	Incorporate equity-conflict scenarios into pediatric pneumonia CBL cases	Improves residents’ ability to design context-sensitive and actionable care plans
Fragmented organizational accountability for health equity	OHL initiatives are frequently disconnected from measurable institutional evaluation systems	Include OHL-Mini-CEX indicators in annual departmental OHL or quality reports	Strengthens institutional accountability for equity-oriented healthcare delivery
Early-stage implementation burden	Faculty adaptation to a new assessment paradigm may initially increase workload	Use phased implementation supported by digital assistance tools and pilot programs	Facilitates gradual integration while minimizing resistance and workflow disruption
Sustainability of educational reform	Short-term educational interventions may fail to achieve long-term institutional change	Establish iterative feedback loops linking educational assessment to policy refinement and organizational process redesign	Supports sustainable transformation toward equity-oriented educational governance

OHL, organizational health literacy; Mini-CEX, Mini-Clinical Evaluation Exercise; CBL, case-based learning.

### 4.3 Summary

This perspective article highlights the potential value of incorporating OHL-oriented assessment into clinical training as one component of broader organizational efforts to advance health equity. It should reach the point-of-care clinical practice—the authentic setting of clinical teaching. Only when health equity principles are embedded into the formative assessment systems that shape residency training can OHL move from institutional policy to professional instinct.

The proposed OHL-Mini-CEX framework provides a practical educational approach for linking health literacy principles with residency assessment. It functions both as an incubator for cultivating future clinicians with heightened awareness of health equity, and as living evidence that healthcare institutions are meeting their organizational responsibilities in the domain of public health. Importantly, the framework remains conceptual and requires empirical validation in diverse clinical settings.

We call upon public health education researchers and clinical teaching administrators to dismantle disciplinary barriers and jointly undertake multicenter empirical studies at this intersection. Such research should verify the real-world effectiveness of this pedagogical model in reducing health inequities in childhood pneumonia outcomes, thereby yielding generalizable solutions to bridge the persistent gap between clinical education and population health.

## Data Availability

The original contributions presented in the study are included in the article/supplementary material, further inquiries can be directed to the corresponding author.
